# Food allergy in patients with confirmed celiac disease

**DOI:** 10.1186/2045-7022-1-S1-P27

**Published:** 2011-08-12

**Authors:** Hadi Peyman, Mohammed Rez Hafezi Ahmadi, Monireh Yaghoubi, Ezatollah Mahmodi, Ali Delpisheh

**Affiliations:** 1 Ilam University of Medical Sciences, Ilam, Islamic Republic of Iran; 2 Ilam University of Medical Sciences, Clinical Pathology, Ilam, Islamic Republic of Iran; 3 Ilam University of Medical Sciences, Clinical Epidemiology, Ilam, Islamic Republic of Iran

## Background

Celiac disease is mediated by the immune system response to ingested gliadin component of gluten present in wheat. Therefore patients with celiac disease have basically sensitive immune systems which can hypersensitive reactions to different food allergens. The current study aimed to assess food allergy in patients with confirmed celiac disease.

## Methods

In this study 25 celiac patients with a mean age of 30.8 ± 13.6 years in Ilam province western Iran were recruited. Approximately 6 ml blood sample was taken from each participant. Food allergy was assessed by EUROIMMUN kit and analyzed by EURO line Scan software (Es). According to Es, patients are categorized into 5 categories including 0 (no specific antibody), 1 (very weak antibodies with no clinical symptoms), 2 (a weak antibody detection/AD and often existing sensitization), 3 (a definite AD), 4 (a strong AD) and 5 (very high antibody titer). The current kit evaluates different food allergy such as fruits, egg, peanut, potato, meat, shellfish, rice and milk.

## Results

Out of 25 participants 68% (n=17) were female and 12% (n=3) were school children. Overall, 68% of cases had a food allergy to mixed shellfish. Of them, 16% (n=4) categorized in class 1, 48% (n=12) in class 2 and 40% (n=10) were in classes between 3 to 5. Four patients (16%) had a food allergy to Sesame.

## Conclusions

Compared to the similar studies amongst Asian populations, celiac patients living in western Iran had a different pattern of food allergy in which peanut and tree nut were created a low level of allergies and inversely allergy with shellfish was more common. Environmental factors and genetic susceptibilities might contribute to these differences.

